# Autophagy promotes hepatic differentiation of hepatic progenitor cells by regulating the Wnt/β-catenin signaling pathway

**DOI:** 10.1007/s10735-018-9808-x

**Published:** 2019-01-02

**Authors:** Zhenzeng Ma, Fei Li, Liuying Chen, Tianyi Gu, Qidi Zhang, Ying Qu, Mingyi Xu, Xiaobo Cai, Lungen Lu

**Affiliations:** Department of Gastroenterology, Shanghai General Hospital, Shanghai Jiao Tong University School of Medicine, Shanghai, 200080 China

**Keywords:** Autophagy, Hepatic progenitor cells, Differentiation, Wnt/β-catenin signaling

## Abstract

Hepatic progenitor cells (HPCs) can be activated when the liver suffers persistent and severe damage and can differentiate into hepatocytes to maintain liver regeneration and homeostasis. However, the molecular mechanism underlying the hepatic differentiation of HPCs is unclear. Therefore, in this study, we aimed to investigate the roles of autophagy and the Wnt/β-catenin signaling pathway during hepatic differentiation of HPCs in vivo and in vitro. First, immunohistochemistry, immunofluorescence and electron microscopy showed that Atg5 and β-catenin were highly expressed in human fibrotic liver and mouse liver injury induced by feeding a 50% choline-deficient diet plus 0.15% ethionine solution in drinking water (CDE diet) for 21 days; in addition, these factors were expressed in CK19-positive HPCs. Second, Western blotting and immunofluorescence confirmed that CK19-positive HPCs incubated in differentiation medium for 7 days can differentiate into hepatocytes and that differentiated HPCs were able to take up ICG and secrete albumin and urea. Further investigation via Western blotting, immunofluorescence and electron microscopy revealed autophagy and the Wnt/β-catenin pathway to be activated during hepatic differentiation of HPCs. Next, we found that inhibiting autophagy by downregulating Atg5 gene expression impaired hepatic differentiation of HPCs and inhibited activation of the Wnt/β-catenin pathway, which was rescued by overexpression of the β-catenin gene. Moreover, downregulating β-catenin gene expression without inhibiting autophagy still impeded the differentiation of HPCs. Finally, coimmunoprecipitation demonstrated that P62 forms a complex with phosphorylated glycogen synthase kinase 3 beta (pGSK3β). Third, in mouse CDE-induced liver injury, immunohistochemistry and immunofluorescence confirmed that downregulating Atg5 gene expression inhibited autophagy, thus impeding hepatic differentiation of HPCs and inhibiting activation of the Wnt/β-catenin pathway. As observed in vitro, overexpression of β-catenin rescued this phenomenon caused by autophagy inhibition, though decreasing β-catenin levels without autophagy inhibition still impeded HPC differentiation. We also found that HPCs differentiated into hepatocytes in human fibrotic liver tissue. Collectively, these results demonstrate that autophagy promotes HPC differentiation by regulating Wnt/β-catenin signaling. Our results are the first to identify a role for autophagy in promoting the hepatic differentiation of HPCs.

## Introduction

The self-renewal capacity of mature hepatocytes contributes to adult liver homeostasis and regeneration; however, the nature of impaired regeneration resulting from chronic liver damage remains unclear (Forbes and Newsome 2016; Chien et al. 2018; López-Luque and Fabregat 2018), and the capacity for self-renewal is overwhelmed following persistent chronic liver injury. In this condition, ductular reaction cells (known as HPCs) expressing SOX9 and cell keratin 19 (CK19) emerge, become activated and then differentiate into functional hepatocytes (Fellous et al. 2009; Passman et al. 2015; Bria et al. 2017). However, the relative contribution of HPCs to parenchymal regeneration remains controversial (Michalopoulos and Khan 2015; Reid 2015; Kopp et al. 2016). The primary reason for this controversy is because the mechanism governing the specific differentiation of HPCs is unclear. Therefore, investigating the mechanism regulating the differentiation of HPCs into functional hepatocytes is urgent.

Autophagy is an evolutionarily conserved ubiquitous process (Thorburn 2018). To decrease internal consumption and prevent toxic accumulation, autophagy-related protein P62 delivers unnecessary and damaged biomacromolecules and organelles in the cytoplasm to lysosomes for degradation via double-membrane autophagosomes (Galluzzi et al. 2014). An increasing number of studies have shown that autophagy plays an important role in stem cell remodeling, regulation of stem cell differentiation and self-renewal (Li et al. 2016; Xue et al. 2016). However, little attention has been devoted to the role of autophagy in the differentiation of HPCs into hepatocytes.

In the absence of Wnt ligands, β-catenin is degraded by ubiquitin proteases after phosphorylation by GSK3β. When Wnt signaling is activated, unphosphorylated β-catenin accumulates in the cytoplasm and then translocates to the nucleus to regulate the expression of target genes (Karner and Long 2017; Steinhart and Angers 2018). The canonical Wnt/β-catenin signaling pathway plays a crucial role in the differentiation and self-renewal of stem cells in various organs (Touboul et al. 2016; Liu et al. 2018; Ihnatovych et al. 2018; He et al. 2018). Recent studies have demonstrated that autophagy regulates the functions of stem cells by the canonical Wnt signaling pathway (Ren et al. 2016; Ozeki et al. 2016). However, the role of the Wnt/β-catenin signaling pathway and its correlation with autophagy in the hepatic differentiation of HPCs remain to be elucidated.

Here, we primarily focus on the role of autophagy and its relationship with the Wnt/β-catenin signaling pathway in the progression of HPC hepatic differentiation.

## Materials and methods

### Patients and liver tissue samples

From September 2015 to December 2017, 50 human liver tissues samples were obtained from the Department of Hepatobiliary Surgery, Shanghai General Hospital, including 25 samples of HBV (Hepatitis B virus)-related fibrotic liver tissue from patients undergoing liver transplant and 25 samples of healthy liver tissue from liver donors. The study was approved by the Ethics Committee of Shanghai General Hospital, and informed consent was obtained from each patient.

### Animals

Five-week-old C57BL/6J male mice were purchased from Sino-British Sippr/BK Laboratory and housed in the Animal Experimental Center of Shanghai First People’s Hospital (Shanghai, China) under specific pathogen-free conditions. Animal protocols were approved by the Chancellor’s Animal Research Committee, and the experiments involving animals adhered to the guidelines set forth by the Shanghai Jiao Tong University School of Medicine (Shanghai, China).

### Liver injury model

Six-week-old male wild-type C57BL/6J mice were fed a choline-deficient, ethionine-supplemented (CDE) diet for 3 weeks (Akhurst et al. 2001). After 3 weeks of the CDE diet, those mice were sacrificed, and hematoxylin and eosin (HE) staining, immunohistochemistry, immunofluorescence staining, and electron microscopy analysis were performed.

### Isolation of HPCs and cell culture

HPCs were isolated from the mice fed a CDE diet for 3 weeks by a modified two-step perfusion protocol (Akhurst et al. 2001), seeded in 90-mm culture dishes, and cultured in complete William’s E medium supplemented with 10% fetal bovine serum (FBS), 2 mM glutamine, 100 U/mL antibiotics, 20 ng/mL epidermal growth factor (EGF, Peprotech), 30 ng/mL human insulin-like growth factor II (IGF-II, GroPep, AUT) and 10 µg/mL insulin (Gibco, USA). After 7 days, clones were selected by local trypsinization in clonal rings, and the selected cells were resuspended in complete medium. After culturing for three generations, purified HPCs were obtained and used for ensuing experiments.

### Induction of hepatic differentiation of HPCs in vitro

HPCs were grown to confluence in complete medium, resuspended with trypsin-EDTA and cultured in differentiation medium for 7 days. The differentiation medium included DMEM/F12 medium (HyClone, SH30023.01B, USA) supplemented with 20% Matrigel (BD, 354230, USA), 40 ng/mL oncostatin M (Sino Biological Inc, 50112-M08H, China), 20 ng/mL hepatocyte growth factor, 10 ng/mL fibroblast growth factor 4 (Sino Biological Inc, China), and 10^−7^ mol/L dexamethasone (Schwartz et al. 2002; Kamiya et al. 2009). To activate the Wnt/β-catenin signaling pathway, HPCs were treated with 4-benzyl-2-methyl-1,2,4-thiadiazolidine-3,5-dione (TDZD-8) (ApexBIO, B1249, USA) at a final concentration of 2.0 µΜ once a day. To inhibite the Wnt/β-catenin signaling pathway, HPCs were treated with recombinant mouse GSK3β protein (Sino Biological, 50650-M07B-50, USA,) at a final concentration of 100 ng/mL once a day.

### Plasmid construct and transient transfection

pGMLV-SC5 RNAi (Genomeditech, Shanghai, China) containing a green fluorescent protein (GFP) reporter was used to express short hairpin RNA (shRNA) targeting the sequences of ATG5 (5′-GCCATCAACCGGAAACTCATG-3′) and β-catenin (5′GCACCATGCAGAATACAAATG-3′), with PGMLV-6395 (vector) and a scramble sequence (5′-TGTTCTCCGAACGTGTCACGT-3′) as a control plasmid. PGMLV-6395 containing a GFP reporter was used to overexpress RNA targeting the sequence of β-catenin (CTCGAGGCCACCGGATCC). Briefly, HPCs were transfected in differentiation medium with shAtg5, shβ-catenin, β-catenin overexpression vector and the corresponding scramble or empty vector using Lipofectamine 3000 reagent (Invitrogen, Carlsbad, CA) according to the manufacturer’s protocol. Following incubation for 48 h, the expression levels of the genes and proteins corresponding to the plasmids transfected were detected by quantitative real-time PCR (qRT-PCR) and Western blotting (WB) on the fifth day.

### Plasmids transfected in vivo

A total of 25 male C57BL/6J mice were randomly divided into a normal diet group (ND, N = 5), blank group (n = 5), shAtg5 group (n = 5), shAtg5 combined with β-catenin overexpression group (n = 5) and shβ-catenin group (n = 5). Plasmid transfection was performed as previously described (Gong et al. 2010). Briefly, shAtg5 plasmid (25 µg), shAtg5 plasmid (25 µg) combined with β-catenin overexpression plasmid (25 µg), and shβ-catenin plasmid (25 µg) were diluted in 150 µl of Opti-MEM medium. Then, each mixture was thoroughly mixed with Lipofectamine 3000 reagent (40 µL) in 150 µL of Opti-MEM medium. The combined 300-µL mixture was incubated at room temperature for 30 min and then injected into mice in each group through the tail vein. This injection was performed at 2:00 pm once every 2 days for a total of ten times.

### Immunohistochemistry (IH)

Liver tissues were fixed with 4% paraformaldehyde, embedded in paraffin after dehydration, and cut into 5-µm sections. The sections then underwent dewaxing, rehydration (> 15 min), repair by sodium citrate antigen (20 min), and quenching in 3% H_2_O_2_ (15 min) and were washed three times with PBS. They were then blocked with antibody with 5% bovine serum albumin (BSA) (30 min) and incubated with the primary antibodies overnight at 4 °C in 1% BSA. Next, the sections were incubated with horseradish peroxidase-conjugated secondary antibodies for 1 h at room temperature and counterstained with hematoxylin, and images were acquired with a light microscope. Information on the primary antibodies is listed in Table 2. Three sections of each liver tissue sample were used for the experiment, five high-powered lens fields were randomly chosen in each section, and the Barnes method was used for immune scoring. The number of expressed cells was analyzed by a quantitative digital image analysis system (Image Pro-Plus, version 6.0). A histogram of the immune score was generated with GraphPad Prism (GraphPad Software Inc., USA, version 8.0).

### Electron microscopy

HPCs and liver tissue were fixed with 2.5% glutaraldehyde in 0.2 M cacodylate buffer (pH 7.4) for 3 h, postfixed with 1% osmium tetroxide for 1.5 h, dehydrated with graded alcohol through propylene oxide, and then embedded in Epon-Araldite resin (Canemco & Marivac, Lakefield, Quebec, Canada). Next, ultrathin sections were stained with uranyl acetate and 0.3% lead citrate and examined under a transmission electron microscope (Toronto, Ontario, Canada). Three sections of each liver tissue were used for the experiment, and a histogram of the number of autophagosomes was generated with GraphPad Prism (GraphPad Software Inc., USA, version 8.0).

### Immunofluorescence (IF)

Liver tissues were fixed with 4% paraformaldehyde, embedded in paraffin, and then cut into 5-µm sections. After antigen retrieval, the specimens were incubated with Tris-EDTA (pH 9.0) for 10 min at 95 °C and 0.1% Triton solution (Sigma-Aldrich), blocked with antibody with TBST solution (TBS-0.1% Tween 20) (Sigma-Aldrich) with 5% BSA, incubated with primary antibody overnight at 4 °C in TBST with 1% BSA, washed twice and incubated with secondary antibodies at room temperature for 1 h. HPCs were cultured in confocal dishes for 7 days, fixed with 4% paraformaldehyde, permeabilized with PBS containing 0.1% Triton X-100, incubated with the primary antibody overnight at 4 °C, incubated with the secondary antibody, and observed under confocal microscopy. Primary antibody information is listed in Table 2. The secondary antibodies were as follows: Alexa Fluor 488 AffiniPure donkey anti-rabbit IgG (H + L) (1:100; Yeasen, China), Alexa Fluor 594 AffiniPure donkey anti-rabbit IgG H + L (1:100; Yeasen, China), Alexa Fluor® 594 goat anti-rabbit IgG H + L (1:200; Abcam, USA), and Alexa Fluor® 594 donkey anti-goat IgG H + L (1:200; Abcam, USA). Images were acquired with a Leica TCS SP8X microscope (Leica, Germany), and five high-powered lens fields were randomly chosen for each section. The number of expressed cells was analyzed by a quantitative digital image analysis system (Image Pro-Plus, version 6.0). Three sections of each liver tissue were used for the experiment, and the HPC immunofluorescence was repeated in three separate experiments.

### Ad-mCherry-GFP-LC3B adenovirus transfection

HPCs were cultured on confocal dishes for 5 days and grown to 40–50% confluence at the time of transfection. After being washed with PBS two times, the cells were transfected with Ad-mCherry-GFP-LC3B adenovirus (Beyotime, C3011, China) at a multiplicity of infection (MOI) of 40 in 200 µL of DMEM/F12 containing 10% FBS, followed by incubation for 48 h at 37 °C. Autophagy was observed under a Leica TCS SP8X microscope (Leica, Germany) and evaluated by calculating the number of yellow and red puncta. The number of expressed cells was analyzed using a quantitative digital image analysis system (Image Pro-Plus, version 6.0).

### Indocyanine green (ICG) uptake and release assay

Briefly, HPCs in 6-well plates were incubated in DMEM/F12 or William’s E medium supplemented with ICG (AAT Bioquest, USA) at a final concentration of 1 mg/mL for 1 h at 37 °C in 5% CO_2_. Next, the DMEM/F12 or William’s E medium was removed, and then HPCs were gently washed three times with PBS. ICG-positive cells were photographed under a microscope (Hitachi, Japan), and the number of expressed cells was analyzed using a quantitative digital image analysis system (Image Pro-Plus, version 6.0).

### Real-time PCR

Total RNA was extracted from HPCs using TRIzol™ reagent (Thermo Fisher, USA) at the indicated times and reverse-transcribed using Hieff™ First Strand cDNA Synthesis Super Mix for RT-qPCR (Yeasen, China). RT-PCR was performed using SYBR® Premix Ex Taq TM (Takara, Dalian, China) under the following conditions: 1 cycle at 95 °C for 2 min, 35 cycles at 95 °C for 15 s and 59 °C for 34 s, and 1 min at 72 °C. GAPDH was used as an internal control to normalize for differences in the amount of total RNA in each sample. The primer sequences are listed in Table 1. All experiments were repeated three separate times, the data were analyzed by QuantStudio™ Real-Time PCR software (Life Technologies, USA, version 1x), and a histogram of relative expression of gene was generated with GraphPad Prism (GraphPad Software Inc., USA, version 8.0).

**Table 1:** Primer sequences for RT-PCR analyses

Target	Forward primer	Reverse primer
Atg5	ATGATTCACGGGATAGAG	GGACAATGCTAATATGAAGA
LC3B	TGATTATAGAGCGATACA	CGTCTGATTATCTTGATG
P62	ATCCCAATGTCAATTTCC	CATCAATGTCAACCTCAA
β-Catenin	ATGGAGGAGATAGTAGAA	CAAACAATGGAATGGTAT
GAPDH	ACCACAGTCCATGCCATCAC	TCCACCACCCTGTTGCTGTA

### Western blotting assay

RIPA lysis buffer (Beyotime, China) containing protease inhibitor cocktail (Sigma-Aldrich, USA) was used for total protein extraction. Equal amounts of protein samples (60 µg) were separated by 10% SDS-PAGE, and the electrophoresed proteins were transferred to PVDF membranes. The membranes were blocked with 5% nonfat milk powder with 0.1% TBST for 1 h and then incubated with primary antibodies at 4 °C for 12 h. Afterward, the membranes were washed three times with TBST and incubated with the corresponding secondary antibody for 1 h. Finally, the membranes were washed three times with TBST; bands were detected with an enhanced chemiluminescence (ECL) (Solarbio, China) system and exposed to X-ray films (LAS MINI 4000, Japan). The relative densities of immunoreactive bands were determined using ImageJ software (National Institute of Health, Bethesda, MD). All experiments were repeated three separate times. Protein expression levels were normalized against GAPDH. Primary antibody information is listed in Table 2.

**Table 2:** Antibodies for WB, IF and co-IP

Antibody	Dilution	Supplier	Product ID
Atg5	1:3000(WB)	Abcam	ab108327
pGSK3β	1:20(IP), 1:1000(WB)	Affinity	AF2016
IgG	1:20(IP)	Abcam	Ab6728
P62	1:3000(WB), 1:100(IF),1:20(IP)	Abcam	ab155686
β-Catenin	1:5000(WB)	Abcam	ab32572
LC3B	1:500(WB)	Proteintech	18725-1-AP
GAPDH	1:10,000 (WB)	Abcam	ab8245
ALB	1:1000(WB), 1:200(IF)	Abcam	ab106582
CK19	1:50,000 (WB), 1:200(IF)	Abcam	ab52625
HNF4α	1:1000(WB), 1:200(IF)	Abcam	ab41898
SOX9	1:1000(WB)	Abcam	ab3697

### Coimmunoprecipitation (Co-IP)

Total protein extracts from HPCs were prepared using Pierce coimmunoprecipitation (Co-IP) kits (Thermo, USA), and protein concentrations were determined using a bicinchoninic acid (BCA) protein quantification kit (Beyotime, China). Coimmunoprecipitation was performed according to the manufacturer’s protocol for the Pierce coimmunoprecipitation kit. Briefly, precleared lysate was prepared using the control agarose resin. Next, immobilized anti-P62 (10 µg/mg lysate), anti-pGSK3 (10 µg/mg lysate) and control IgG antibodies (10 µg/mg lysate) were added to the amino link plus coupling resin, and 400 µg of precleared lysate was incubated with different immobilization antibodies overnight at 4 °C. Finally, after being washed with 60–70 µL of elution buffer, the immunoprecipitates were boiled for 10 min and then assessed by WB. Information on the primary antibodies is listed in Table 2.

### Albumin (ALB) production in vitro

The supernatants of HPCs cultured in 24-well dishes were collected on the seventh day and frozen at − 20 °C until assay. The supernatants were assayed for ALB production using a quantitative enzyme-linked immunosorbent assay kit (Albumin ELISA kits, CUSABIO BIOTECH, China, CSB-E13878m) according to the manufacturer’s recommendations. A histogram of the ALB concentration was generated with GraphPad Prism (GraphPad Software Inc., USA, version 8.0).

### Urea production in vitro

HPCs were incubated with 0.5 mL of medium containing 5 mM NH_4_Cl (Sigma) for 24 h in 5% CO_2_ at 37 °C on the seventh day. Next, the supernatant was collected, and urea concentrations were measured using urea ELISA kits (SenBeiJia Biological, China, SBJ-M0080) according to the manufacturer’s protocol. A histogram of the urea concentration was generated with GraphPad Prism (GraphPad Software Inc., USA, version 8.0).

### Statistical analysis

Data are presented as means ± standard deviations (SDs). All statistical analyses were performed using SPSS 20.0 software (IBM SPSS; Armonk, NY, USA). Comparisons between two groups were analyzed using an independent t-test; differences among groups were performed by one-way ANOVA. *P* < 0.05 indicated a statistically significant result.

## Results

### Atg5 and β-catenin were coexpressed in CK19-positive ductular reaction cells in human fibrotic liver tissue

**Fig. 1:**
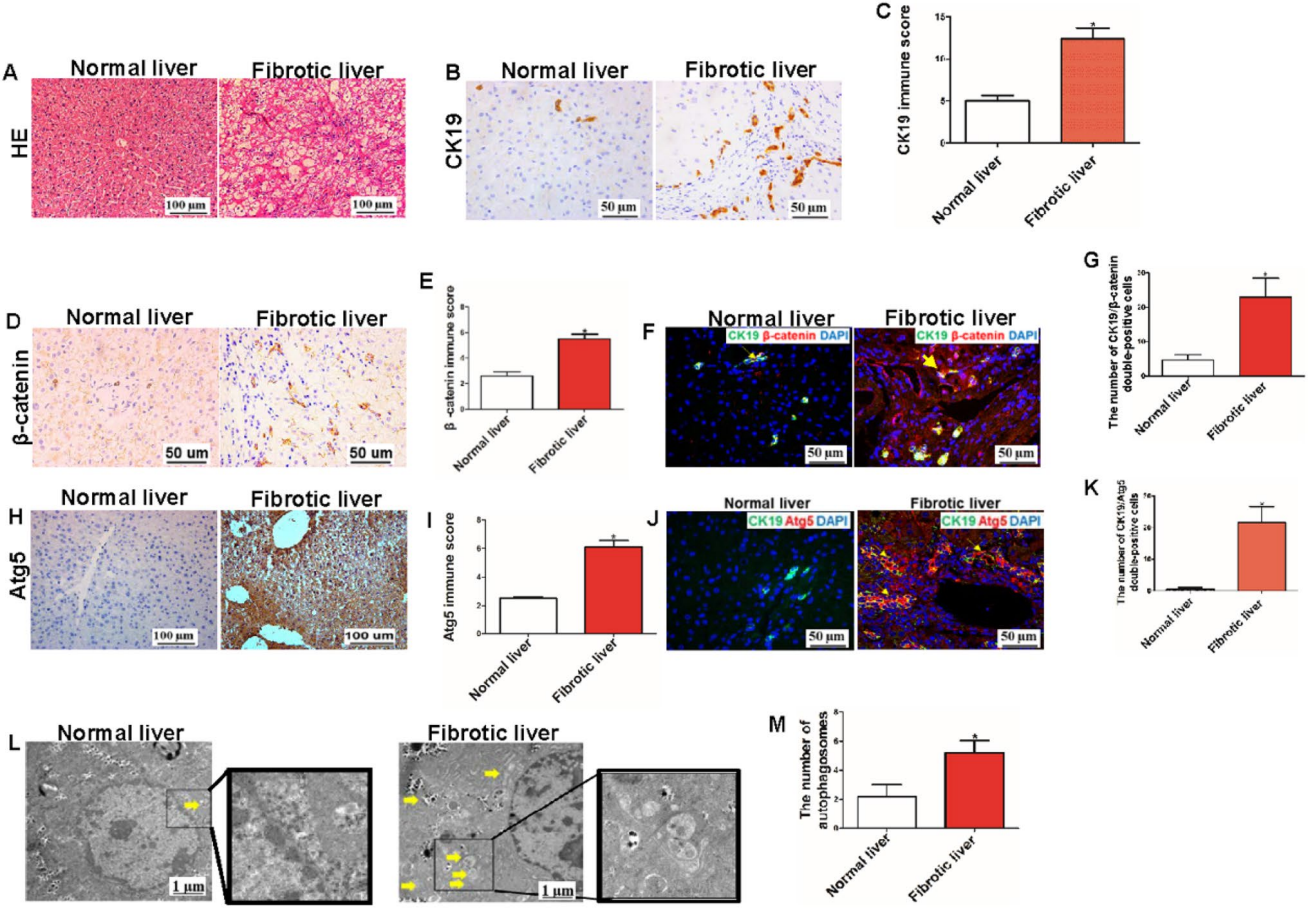
β-Catenin and Atg5 were expressed in CK19-positive ductular reaction cells in human fibrotic liver. **a** HE staining in human fibrotic liver (scale bar = 100 µm, n = 25 per group). **b, c** Immunochemical staining and histogram presenting quantification of the immune score for CK19 in human liver tissue (*P < 0.05 compared with control group, scale bar = 50 µm, n = 25 per group). **d, e** Immunochemical staining and histogram presenting quantification of the immune score for β-catenin (*P < 0.05 compared with control group, scale bar = 50 µm, n = 25 per group). **f, g** Immunofluorescence staining for CK19 and β-catenin and histogram showing quantification of β-catenin/CK19 double-positive ductular reaction cells (yellow arrow) (*P < 0.05 compared with control group, scale bar = 50 µm, n = 25 per group). **h, i** Immunochemical staining and immune score for Atg5 (*P < 0.05 compared with control group, scale bar = 100 µm, n = 25 per group). **j, k** Immunofluorescence staining for CK19 and Atg5; Atg5 expression in CK19-positive ductular reaction cells (yellow arrow) (*P < 0.05 compared with control group, scale bar = 50 µm, n = 25 per group). **l, m** Autophagosomes (arrowheads) were observed in control and fibrotic liver under an electron microscope. Histogram showing quantification of autophagosomes (*P < 0.05 compared with control group, scale bar = 1 µm, n = 25 per group)

Previous studies have demonstrated that ductular reaction cells appear during chronic liver injury and differentiated into hepatocytes, thus maintaining hepatic homeostasis (Fellous et al. 2009; Passman et al. 2015; Bria et al. 2017). In our study, we first confirmed that severe liver injury existed in human chronic liver injury (Fig. 1a) and that bile ductular cells were highly expanded (Fig. 1b, c). Moreover, β-catenin expression was higher in the fibrotic liver than in the normal liver (Fig. 1d, e). Following further investigation, β-catenin appeared not only in cholangiocytes in the normal liver but also in CK19-positive ductular reaction cells in the fibrotic liver, and the number of β-catenin/CK19 double-positive cells in fibrotic liver tissue was higher (Fig. 1f, g). To assess autophagy activation, IH suggested that the expression level of Atg5 was significantly increased in fibrotic liver tissue (Fig. 1h, i). Next, an IF assay demonstrated that Atg5 was not only present in the liver parenchyma but also appeared in CK19-positive ductular reaction cells and that Atg5/CK19 double-positive cells were more numerous in the fibrotic liver (Fig. 1j, k). In addition, electron microscopy confirmed that the number of autophagosomes in fibrotic liver tissue was higher than that in normal liver tissue (*P* < 0.05, Fig. 1l, M).

These data indicate that autophagy and the Wnt/β-catenin signaling pathway were activated in human chronic liver injury and that they coexisted in CK19-positive ductular reaction cells.

### Atg5 and β-catenin were also coexpressed in mouse CK19-positive ductular reaction cells after treatment with a CDE diet for 21 days

**Fig. 2:**
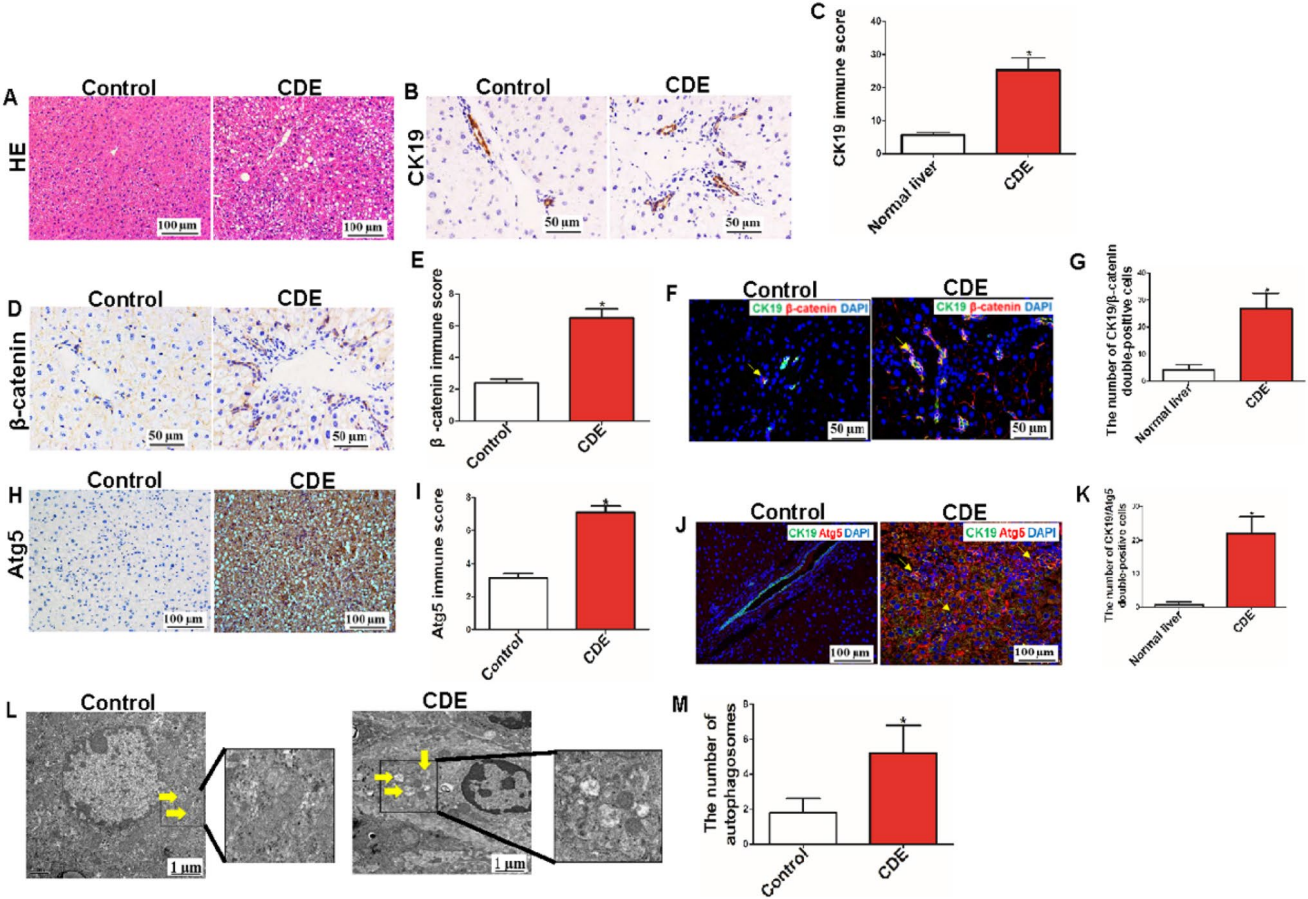
β-catenin and Atg5 were expressed in the CK19-positive ductular reaction cells in mouse liver after CDE exposure. **a** HE staining of C57BL/6 mouse liver tissue (scale bar = 100 µm, n = 5 per group). **b, c** Immunochemical staining and immune score for CK19 (*P < 0.05 compared with control group, scale bar = 50 µm, n = 25 per group). **d, e** Immunochemical staining and immune score for β-catenin (*P < 0.05 compared with control group, scale bar = 50 µm, n = 5 per group). **f, g** Immunofluorescence staining for CK19 and β-catenin; histogram showing quantification of CK19/β-catenin double-positive cells; β-catenin was expressed in CK19-positive ductular reaction cells (yellow arrow) (*P < 0.05 compared with control group, scale bar = 50 µm, n = 25 per group). **h, i** Immunochemical staining and immune score for Atg5 (*P < 0.05 compared with control group, scale bar = 100 µm, n = 5 per group). **j, k** Immunofluorescence staining for CK19 and Atg5; histogram showing quantification of CK19/Atg5 double-positive cells, Atg5 was expressed in CK19-positive ductular reaction cells (yellow arrow) (*P < 0.05 compared with control group, scale bar = 100 µm, n = 5 per group). **l, m** Autophagosomes (arrowheads) were observed and quantified in control and CDE-damaged liver tissue (*P < 0.05 compared with control group, scale bar = 1 µm, n = 5 per group)

To further investigate whether autophagy and the Wnt/β-catenin signaling pathway also coexisted in mouse CK19-positive ductular reaction cells during chronic liver injury, C57BL/6J mice were treated with a CDE diet for 21 days and then sacrificed. First, we found that severe liver injury and inflammatory reactions were present with CDE-induced liver injury (Fig. 2a). Furthermore, ductular reaction cells were highly expanded (Fig. 2b, c), and β-catenin was highly expressed in the CDE liver (Fig. 2d, e). Second, to further uncover the relationship between CK19 and β-catenin, IF staining for CK19 and β-catenin was applied to liver tissue. The results revealed that β-catenin was not only expressed in normal cholangiocytes but also in CK19-positive ductular reaction cells and that the number of β-catenin/CK19 double-positive cells in fibrotic liver tissue was higher than that in healthy liver tissue (Fig. 2f, g). Third, IH suggested that expression of Atg5 was significantly increased in CDE liver tissue (Fig. 2h, i). IF assays also demonstrated that Atg5 was significantly increased in CDE-induced liver injury and that Atg5 was present not only in the liver parenchyma but also in CK19-positive ductular reaction cells. In addition, Atg5/CK19 double-positive cells were more numerous in CDE-induced liver injury (Fig. 1j, k). Finally, electron microscopy showed that the number of autophagosomes was higher in the CDE-damaged liver (Fig. 1l, m).

These data indicate that autophagy and the Wnt/β-catenin signaling pathway were activated in mouse CDE liver injury and that they coexisted in CK19-positive ductular reaction cells.

### Autophagy was highly activated during hepatic differentiation of HPCs in vitro

**Fig. 3:**
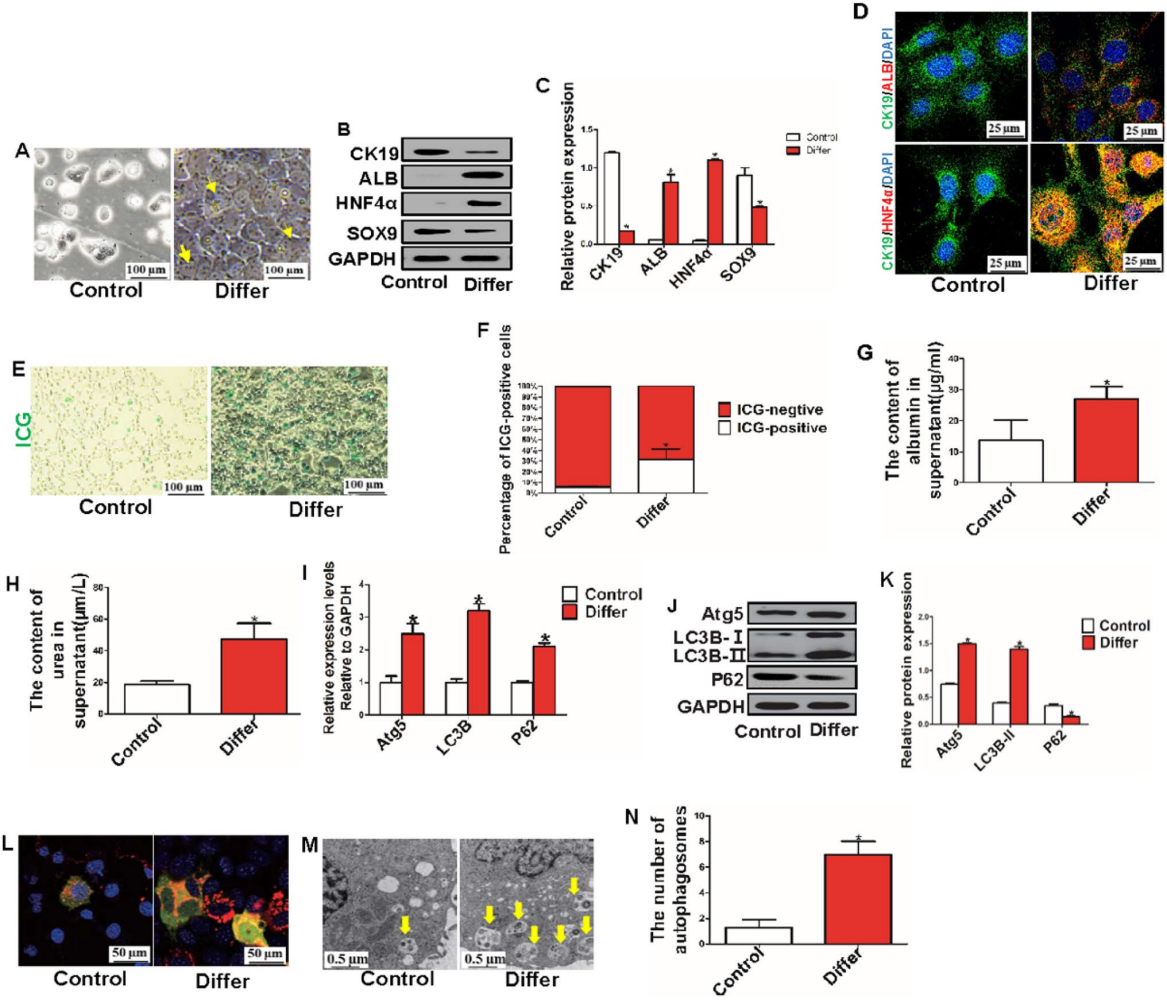
Increased activation of autophagy occurred in the progression of hepatic differentiation of HPCs in vitro. **a** Representative phase contrast microscopy image in control and differentiated HPCs (scale bar = 100 µm, n = 3). **b, c** Expression of HPC (CK19 and SOX9)- and hepatocyte (ALB and HNF4α)-related proteins was analyzed in the control and differentiated groups (*P < 0.05 compared with control group, repeated in three experiments, n = 3 per experiment). **d** Immunofluorescence staining for CK19, ALB and HNF4α was performed in the control and differentiated groups (scale bar = 25 µm, n = 3). **e, f** ICG uptake assay. HPCs with a green-stained nucleus were considered positive (*P < 0.05 compared with control group, scale bar = 100 µm, n = 3). **g, h** ALB and urea production in the supernatant were detected by ELISA kits (*P < 0.05 compared with control group, n = 3). **i** Relative expression of autophagy-related genes was detected by RT-PCR (*P < 0.05 compared with control group, data are the mean ± SD, n = 3). **j, k** Relative expression of autophagy-related proteins was detected in the control and differentiated groups (*P < 0.05 compared with control group, data are the mean ± SD, repeated in three experiments, n = 3 per experiment). **l** Representative images of HPCs transfected with Ad-mCherry-GFP-LC3B adenovirus (scale bar = 50 µm, n = 3). **m, n** Autophagosomes (arrowheads) were observed in control and differentiated HPCs under an electron microscope (*P < 0.05 compared with control group, scale bar = 0.5 µm, n = 3)

To investigate the effect of autophagy and the Wnt/β-catenin signaling pathway on the physiological function of CK19-positive HPCs, a model of hepatic differentiation of HPCs was established in vitro, in which HPCs were induced in differentiation medium for 7 days. First, the morphology of differentiated HPCs changed from oval to polygon in shape, and partially differentiated HPCs had a double nucleus (Fig. 3a). WB found that differentiated HPCs exhibited downregulated CK19 and SOX9 expression and upregulated ALB and HNF4α expression (Fig. 3b, c). Second, IF staining was used to trace the hepatic differentiation of HPCs in vitro, and the results showed that differentiated HPCs were double-positive for CK19/ALB or CK19/HNF4α but that HPCs in the control group were mainly CK19-positive, indicating that HPCs can differentiate into hepatocytes in differentiation medium for days (Fig. 3d). Third, mature hepatocytes have the ability to take up ICG and secrete ALB and urea; thus, to confirm a similar ability in differentiated HPCs, HPCs were treated with ICG (1 mg/mL). We found that differentiated HPCs acquired more ICG (Fig. 3e, f) and that differentiated HPCs produced more ALB and urea (Fig. 3g, h). Fourth, autophagy-related genes and proteins in HPCs were detected by RT-PCR and WB, respectively. The expression levels of Atg5, LC3B and P62 genes in the differentiation groups were higher than those in the control group (Fig. 3i). Moreover, the expression levels of Atg5 and LC3B-II proteins were increased, but that of P62 was decreased in differentiated HPCs, indicating that autophagy was activated during hepatic differentiation of HPCs on the seventh day (Fig. 3j, k). To further identify increased activation of autophagy during hepatic differentiation, HPCs were transfected with Ad-mCherry-GFP-LC3B adenovirus on the fifth day and incubated for 48 h. Red dots (mCherry fluorescence) indicate autophagosomes that had fused with lysosomes, and yellow dots (merged mCherry and GFP fluorescence) represent autophagosomes that were not fused with lysosomes. Thus, activation of autophagy was indicated through an increase in both red and yellow dots, and the results showed that autophagy was highly activated in differentiated HPCs (Fig. 3l). In addition, electron microscopy showed that the number of autophagosomes was also significantly increased in the differentiated HPCs (Fig. 3m, n).

These data suggest that CK19-positive HPCs can differentiate into hepatocytes and that autophagy is highly activated during this progression.

### Inhibition of autophagy impeded hepatic differentiation of HPCs in vitro

**Fig. 4:**
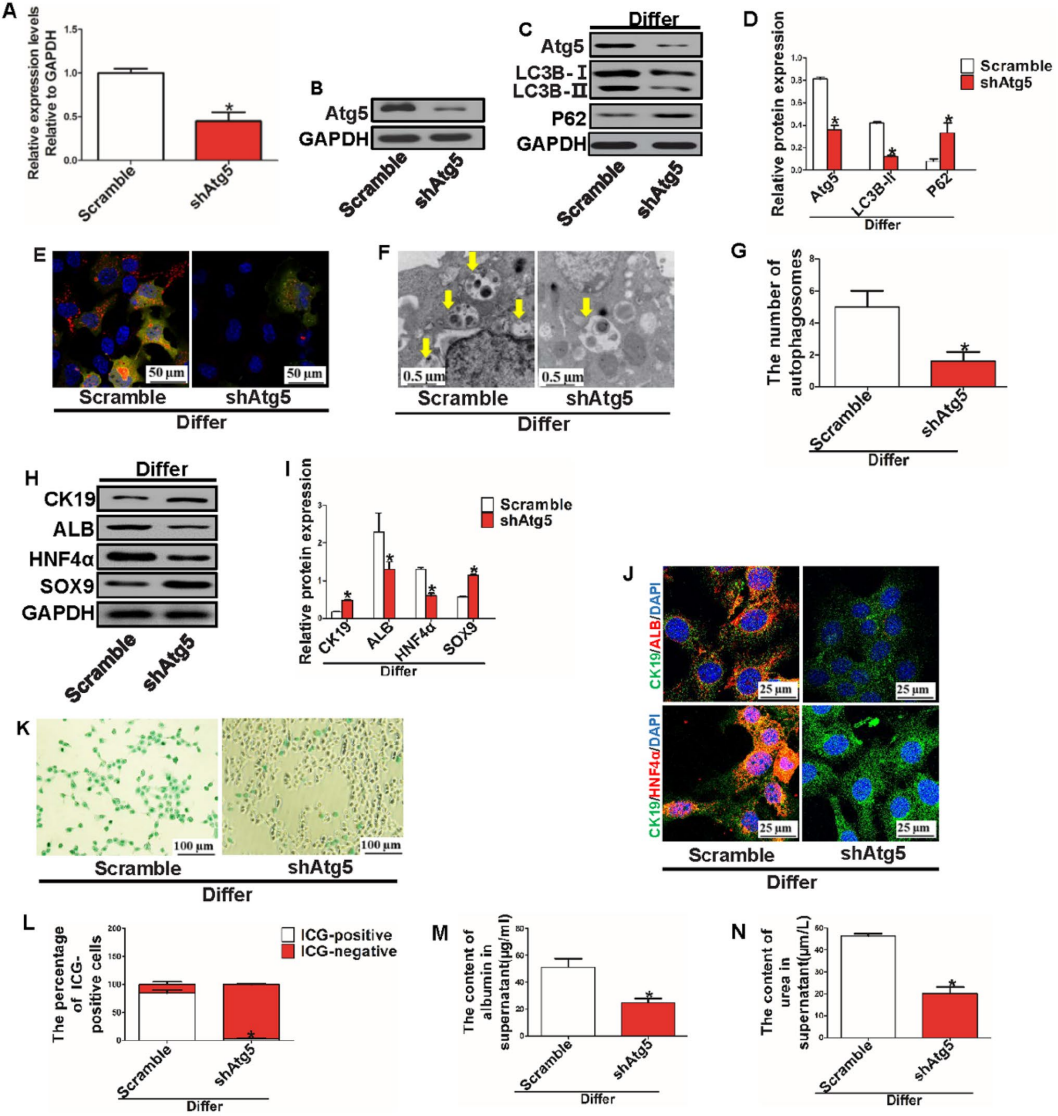
Downregulation of Atg5 impeded the hepatic differentiation of HPCs in vitro. **a, b** Expression of Atg5 mRNA and protein was detected by RT-PCR and WB (*P < 0.05 compared with scramble group, data are the mean ± SD, repeated in three experiments, n = 3 per experiment). **c, d** Relative expression of autophagy-related protein was detected by WB (*P < 0.05 compared with scramble group, data are the mean ± SD, repeated in three experiments, n = 3 per experiment). **e** Representative images of autophagy of HPCs transfected with Ad-mCherry-GFP-LC3B adenovirus in different groups (scale bar = 50 µm, n = 3). **f, g** Autophagosomes (arrowheads) were observed under electron microscopy (*P < 0.05 compared with scramble group, data are the mean ± SD, scale bar = 0.5 µm, n = 3). **h, i** WB analysis showed expression of HPC (CK19 and SOX9)- and hepatocyte (ALB and HNF4α)-related proteins in different groups (*P < 0.05 compared with scramble group, data are the mean ± SD, repeated in three experiments, n = 3 per experiment). **j** Immunofluorescence staining for CK19, ALB and HNF4α was performed in different groups; endogenous expression and distribution of ALB, CK19 and HNF4α were observed under a confocal microscope (scale bar = 25 µm, n = 3). **k, l** ICG uptake assay. HPCs with a green-stained nucleus were considered positive, which indicated differentiated HPCs (*P < 0.05 compared with scramble group, data are the mean ± SD, scale bar = 100 µm, n = 3). **m, n** ALB and urea production in the supernatant were detected by ELISA kits in different groups (*P < 0.05 compared with scramble group, n = 3)

To further investigate the effect of autophagy on the hepatic differentiation of HPCs, HPCs were treated with scramble nucleotides and shAtg5 plasmids in differentiation medium on the second day and allowed to incubate for 5 days. Total RNA and protein were extracted from HPCs, and RT-PCR and WB revealed that the shAtg5 plasmid effectively inhibited Atg5 gene and protein expression (Fig. 4a, b). Furthermore, we found that the shAtg5 plasmid effectively inhibited autophagy activation (Fig. 4c, d); the Ad-mCherry-GFP-LC3B assay also confirmed that autophagy activation was inhibited by the shAtg5 plasmid (Fig. 4e). Finally, electron microscopy showed that the number of autophagosomes was dramatically reduced with downregulation of Atg5 (Fig. 4f, g), indicating that autophagy activation can effectively be inhibited by the shAtg5 plasmid. Second, to investigate the effect of autophagy inhibition on hepatic differentiation of HPCs, WB was performed in different groups. The results indicated that autophagy inhibition impaired hepatic differentiation of HPCs (Fig. 4h, i); IF showed that without inhibition of autophagy, HPCs were CK19/ALB or CK19/HNF4α double-positive, but that when autophagy was inhibited, HPCs were mainly CK19-positive, also indicating that hepatic differentiation of HPCs was inhibited with autophagy inhibition (Fig. 4j). Finally, the ICG absorption assay confirmed that the number of ICG-positive cells was significantly decreased with autophagy inhibition (Fig. 4k, l). In addition, the levels of ALB and urea in the supernatant were decreased in HPCs when autophagy was inhibited (Fig. 4m, n).

These data show that autophagy inhibition can impair hepatic differentiation of HPCs.

### The Wnt/β-catenin signaling pathway was activated during the progression of hepatic differentiation of HPCs in vitro, and inhibition of autophagy downregulated β-catenin protein expression

**Fig. 5:**
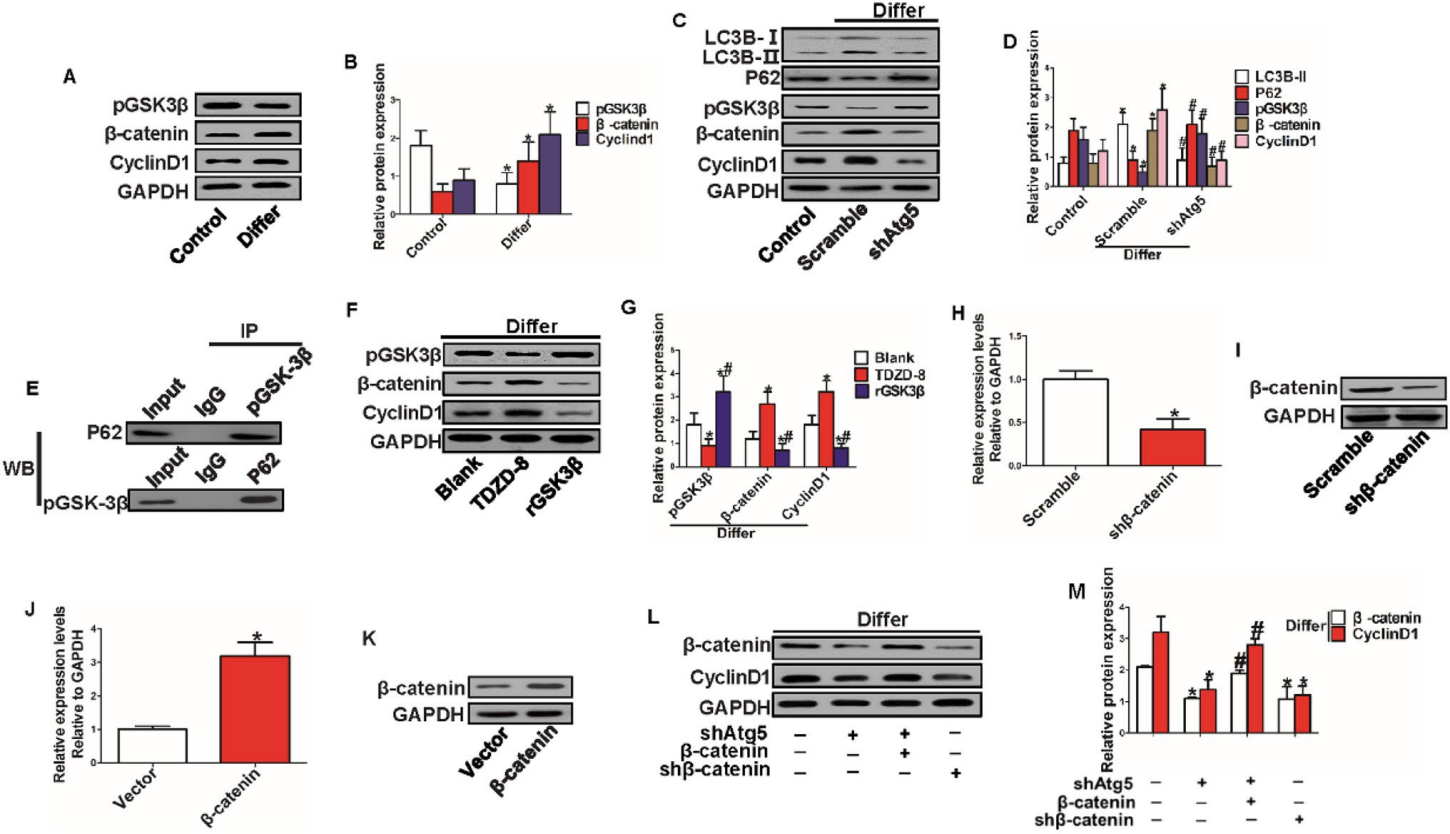
Inhibition of autophagy by downregulation of Atg5 suppressed the expression of β-catenin protein in the progression of hepatic differentiation of HPCs in vitro. **a, b** WB analysis of the expression level of Wnt/β-catenin signaling pathway-related proteins (*P < 0.05 compared with control group, data are the mean ± SD, repeated in three separate experiments, n = 3 per experiment). **c, d** Total protein was extracted from HPCs in different groups at 7 days, and expression of LC3B-1, LC3B-II, P62, pGSK3β, β-catenin and CyclinD1 was detected by WB. Histogram showing quantification of WB (*P < 0.05 compared with control group, *#P* < 0.05 compared with scramble group, data are the mean ± SD, repeated in three separate experiments, n = 3 per experiment). **e** Cell lysates of HPCs were coprecipitated with anti-pGSK3β and anti-p62 antibodies and normal IgG as a negative control antibody and then detected by P62 and pGSK3β antibodies; 20% of total cell lysates were used as input. **f, g** WB detected the effect of TDZD-8 (GSK3β-specific inhibitor) and rGSK3β (recombinant mouse GSK3β protein) on the expression of β-catenin and CyclinD1 protein in HPCs in differentiated medium for 7 days; histogram showing quantification of WB (*P < 0.05 compared with Blank group, *#P* < 0.05 compared with TDZD-8 group, data are the mean ± SD, repeated in three separate experiments, n = 3 per experiment). **h, i** RT-PCR and WB analyses detected the effect of shβ-catenin on the expression of β-catenin in HPCs (*P < 0.05 compared with scramble group, data are the mean ± SD, repeated in three experiments, n = 3 per experiment). **j, k** RT-PCR and WB analyses detected the effect of overexpression of β-catenin on the expression of β-catenin in HPCs (*P < 0.05 compared with vector group, data are the mean ± SD, repeated in three experiments, n = 3 per experiment). **l, m** WB was used to detect the expression levels of β-catenin and CyclinD1 in HPCs in different groups;, and quantification of β-catenin and CyclinD1 (*P < 0.05 compared with group without any treatment, #*P* < 0.05 compared with shAtg5 group, data are the mean ± SD, repeated in three experiments, n = 3 per experiment)

First, we investigated whether the Wnt/β-catenin signaling pathway was activated during the hepatic differentiation of HPCs. Interestingly, we found that the Wnt/β-catenin signaling pathway was activated in differentiated HPCs (Fig. 5a, b). Second, we downregulated autophagy activation by inhibiting the Atg5 gene. The results show that the Wnt/β-catenin signaling pathway was activated with autophagic activation and vice versa; in addition, pGSK3β, the phosphorylated form of GSK3β, inhibits activation of the Wnt/β-catenin pathway, and the expression level of pGSK3β and the activation level of autophagy showed opposite tendencies, indicating that the Wnt/β-catenin signaling pathway was activated or inhibited with autophagy activation or inhibition, respectively (Fig. 5c, d). Third, a co-IP assay showed that P62, a protein that can deliver molecules for degradation during the process of autophagy, formed a complex with pGSK3β (Fig. 5e). These data indicated that the autophagy-activated Wnt/β-catenin signaling pathway might be mediated by the degradation of pGSK3β. Next, we treated HPCs with a GSK3β inhibitor (TDZD-8) at a final concentration of 2.0 µM and activator (recombinant mouse GSK3β protein) at 100 ng/mL once a day in differentiation medium for 7 days. The results showed that TDZD-8 downregulated the expression of pGSK3β and upregulated the expression of β-catenin and CyclinD1. Compared to TDZD-8, rGSK3β showed the opposite effect on the Wnt/β-catenin signaling pathway, indicating that β-catenin was the downstream target of pGSK3β (Fig. 5f, g). β-catenin plays a pivotal central role in the activation of the Wnt/β-catenin signaling pathway. Therefore, we treated HPCs with shβ-catenin and the corresponding scramble RNA, as well as with β-catenin overexpression and corresponding empty vector plasmids. The results demonstrated that shβ-catenin could effectively inhibit and β-catenin overexpression could effectively increase the expression of β-catenin (Fig. 5h–k). Finally, even under suppression of the activation of the Wnt/β-catenin signaling pathway by inhibition of autophagy, β-catenin overexpression also increased the expression of β-catenin and CyclinD1, but downregulation of β-catenin and CyclinD1 by shβ-catenin without inhibition of autophagy showed no significant difference from β-catenin and CyclinD1 downregulation induced via inhibition of autophagy alone (Fig. 5l, m).

These data suggest that the canonical Wnt/β-catenin signaling pathway can be regulated by autophagy during the progression of hepatic differentiation of HPCs.

### Overexpression of β-catenin attenuated the impairment of hepatic differentiation of HPCs induced by suppressing autophagy activation

**Fig. 6:**
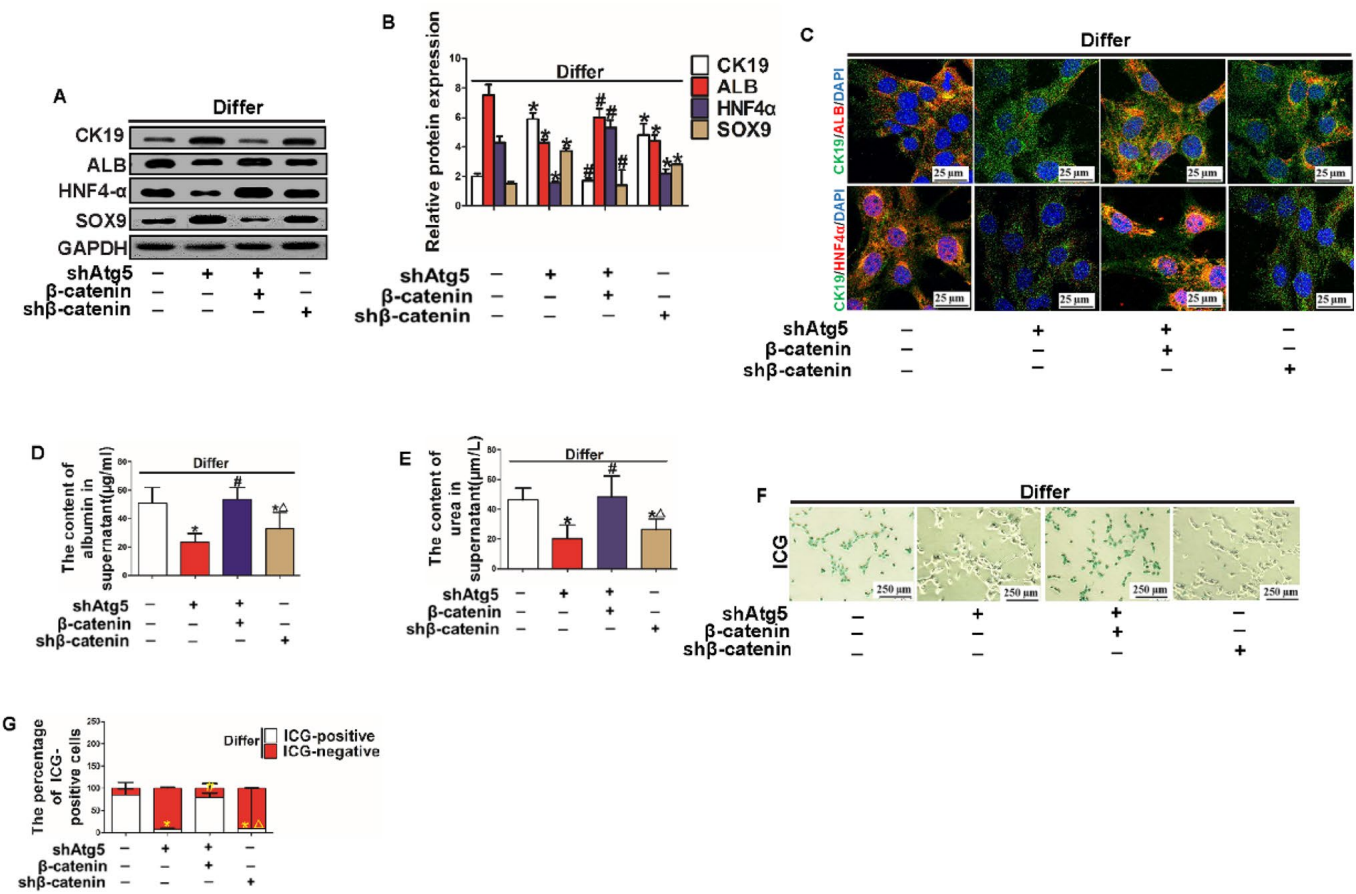
β-catenin overexpression attenuated the impairment in hepatic differentiation of HPCs induced by suppressing autophagy activation. **a, b** WB analysis showed the expression levels of HPC markers (CK19 and SOX9) and hepatocyte markers (ALB and HNF4α) in different groups. Representative quantitative results of WB (*P < 0.05 compared with group without any treatment, #*P* < 0.05 compared with shAtg5 group, data are the mean ± SD, repeated in three experiments, n = 3 per experiment). **c** Immunofluorescence staining for CK19, ALB and HNF4α was performed for each group and observed under a confocal microscope (scale bar = 25 µm, n = 3). **d, e** ALB and urea secretion in the supernatant were detected by ELISA in different groups (*P < 0.05 compared with group without any treatment, #*P* < 0.05 compared with shAtg5 group, ∆P < 0.05 compared with shAtg5 combined with β-catenin overexpression group, data are the mean ± SD, n = 3). **f, g** ICG uptake assay. HPCs with a green-stained nucleus were the positively stained cells; quantification of ICG-positive cells in different groups (*P < 0.05 compared with group without any treatment, #*P* < 0.05 compared with shAtg5 group, *∆P* < 0.05 compared with shAtg5 combined with β-catenin overexpression group, data are the mean ± SD, scale bar = 250 µm, n = 3)

First, to further investigate whether autophagy regulated the Wnt/β-catenin signaling pathway and then influenced the hepatic differentiation of HPCs, WB was performed to investigate the expression levels of CK19, ALB, HNF4α and SOX9 in HPCs induced to hepatic differentiation for 7 days. The results showed that autophagy inhibition impaired hepatic differentiation of HPCs but that this impairment was rescued by overexpression of β-catenin. Moreover, even without autophagy inhibition, downregulation of the expression of β-catenin sufficiently inhibited hepatic differentiation of HPCs, indicating that the Wnt/β-catenin pathway was regulated by autophagy and then influenced HPC hepatic differentiation (Fig. 6a, b). Second, IF staining further confirmed that overexpression of β-catenin rescued the impairment of hepatic differentiation of HPCs induced by inhibition of autophagy. Even when autophagy was inhibited, overexpression of β-catenin increased the number of CK19/ALB or CK19/HNF4α double-positive cells; however, downregulation of β-catenin without inhibition of autophagy still impaired hepatic differentiation (Fig. 6c). Third, further investigation of the secretion function of HPCs revealed that the ability of differentiated HPCs to secrete ALB and urea was inhibited by autophagy inhibition but that this phenomenon was rescued by overexpression of β-catenin. Interestingly, downregulating β-catenin without inhibiting autophagy still impaired ALB and urea secretion (Fig. 6d, e). Finally, the ICG absorption assay further confirmed that the absorptive function of differentiated HPCs was impeded by autophagy suppression but that overexpression of β-catenin could attenuate the impedance (Fig. 6f, g).

These data indicate that autophagy promotes hepatic differentiation of HPCs by activating the Wnt/β-catenin signaling pathway.

### Autophagy regulated hepatic differentiation of HPCs by governing the Wnt/β-catenin signaling pathway in vivo

**Fig. 7:**
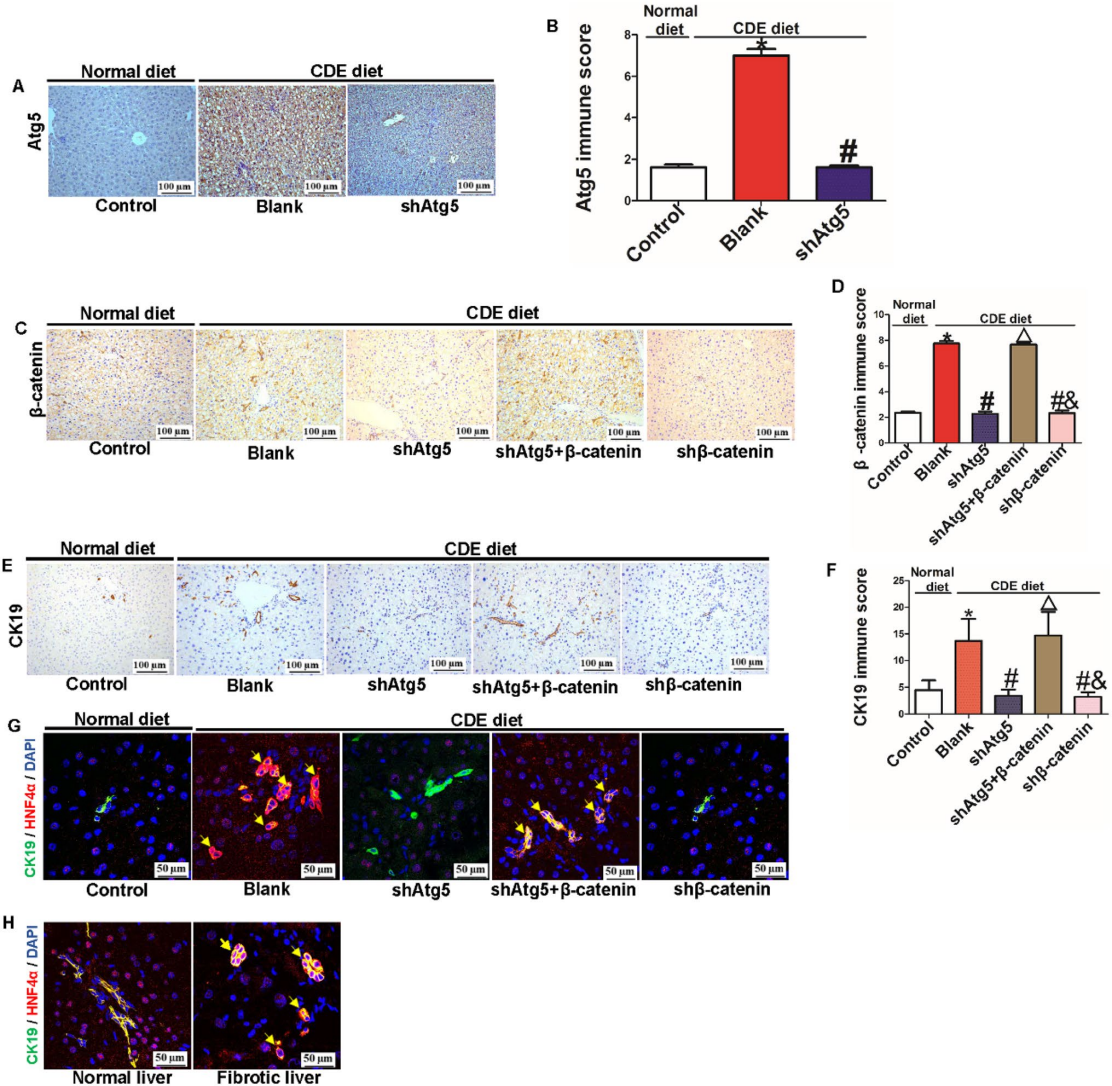
The effect of autophagy on the hepatic differentiation of HPCs in vivo. **a, b** Immunochemical staining for Atg5 and quantitative results of the Atg5 protein score in mouse liver tissue (*P < 0.05 compared with control group, #*P* < 0.05 compared with Blank group, data are the mean ± SD, scale bar = 100 µm, n = 5). **c, d** Immunochemical staining and immune score for β-catenin in different mouse liver tissues (*P < 0.05 compared with control group, #*P* < 0.05 compared with Blank group, Δ*P* < 0.05 compared with shAtg5 group, *&P* < 0.05 compared with shAtg5 combined with β-catenin overexpression group, data are the mean ± SD, scale bar = 100 µm, n = 5). **e, f** Immunochemical staining and quantitative results for CK19 in mouse liver tissue (*P < 0.05 compared with control group, #*P* < 0.05 compared with Blank group, Δ*P* < 0.05 compared with shAtg5, *&P* < 0.05 compared with shAtg5 combined overexpression β-catenin group, data are the mean ± SD, scale bar = 100 µm, n = 5). **g** Immunofluorescence staining for CK19 and HNF4α was performed in different mouse liver tissues, and endogenous expression and distribution of CK19 and HNF4α were observed under a confocal microscope, scale bar = 50 µm. **h** Immunofluorescence staining for CK19 and HNF4α was performed in human liver tissue, and endogenous expression and distribution of CK19 and HNF4α were observed under a confocal microscope, scale bar = 50 µm

To further investigate the influence of autophagy on hepatic differentiation of HPCs by regulating β-catenin in vivo, we treated mice with a CDE diet and then injected shAtg5, shβ-catenin, β-catenin overexpression, and shAtg5 combined with overexpression β-catenin plasmid via the mouse tail vein on the next day. The injection was performed once every 2 days for a total of ten times. The results indicated that autophagy was activated in CDE-induced injury liver (blank group), but autophagy was effectively suppressed by downregulating the expression of the Atg5 gene (Fig. 7a, b). Next, we found that the expression of β-catenin was increased with liver injury but decreased with autophagy inhibition; however, overexpression of β-catenin rescued the downregulation of β-catenin caused by autophagy inhibition. Despite the activation of autophagy, the downregulation of β-catenin also effectively decreased the expression of β-catenin (Fig. 7c, d). Furthermore, we found that autophagy inhibition by downregulating Atg5 gene expression decreased the ductular reaction, but this phenomenon was rescued by overexpression of β-catenin; however, even when β-catenin was downregulated without inhibiting autophagy, the ductular reaction was also decreased, which was similar to the effect induced by the inhibition of autophagy alone (Fig. 7e, f). Finally, we explored whether CK19-positive HPCs can differentiate into hepatocytes in humans and mice with chronic liver injury. IF staining for CK19 and HNF4α was performed in liver tissues from different groups. Remarkably, HNF4α and CK19 biphenotypic cells were detected in CDE liver tissue, indicating that CK19-positive HPCs can differentiate into hepatocytes in chronic liver injury induced by CDE diet treatment. However, this differentiation was impeded by autophagy inhibition. Overexpression of β-catenin restored the hepatic differentiation impairment induced by inhibiting autophagy; however, even without autophagy inhibition, the downregulation of β-catenin also effectively inhibited hepatic differentiation of HPCs (Fig. 7g). Interestingly, we also found hepatic differentiation of HPCs in human chronic liver injury (Fig. 7h).

These data show that autophagy can regulate hepatic differentiation of HPCs by regulating the Wnt/β-catenin signaling pathway in vivo.

## Discussion

Ductular reactions are encountered in a variety of chronic liver diseases, and they are also called oval cell proliferation in rodents (Gjymishka et al. 2016; Khambu et al. 2018). Ductular reactions are formed by a transit-amplifying population (known as HPCs) derived from hepatobiliary progenitor cells, which express biliary markers such as SOX9 and CK19 (Español-Suñer et al. 2012). Although recent studies have shown that HPCs can differentiate into hepatocytes, their contribution to the regeneration of liver parenchyma remains controversial (Fellous et al. 2009; Passman et al. 2015; Bria et al. 2017). The main reason for this controversy is that few studies have focused on the mechanism underlying the hepatic differentiation of HPCs. In this study, we first confirmed that autophagy and Wnt/β-catenin were activated in the human fibrotic liver and in mouse CDE-induced liver injury and that they coexisted in CK19-positive ductular reaction cells. Second, we demonstrated that autophagy and the Wnt/β-catenin signaling pathway were activated during hepatic differentiation of CK19-positive HPCs in vitro. Next, we found that autophagy inhibition suppressed activation of the Wnt/β-catenin signaling pathway and then impaired the hepatic differentiation of HPCs. Further investigation revealed that P62 could form a complex with pGSK3β and that regulation of the Wnt/β-catenin signaling pathway by autophagy might degrade pGSK3β through P62. Finally, we confirmed that autophagy regulated the hepatic differentiation of CK19-positive HPCs through the Wnt/β-catenin signaling pathway in the CDE mouse model of liver injury.

Recent investigations have confirmed that autophagy plays a pivotal role in the progression of chronic liver disease (Wang et al. 2017; Liu et al. 2018). However, few studies have focused on the relationship between autophagy and the physical function of CK19-positive HPCs. In this study, we found the autophagy-related protein Atg5 to be highly expressed in human fibrotic liver tissue and in the mouse CDE liver injury model and was present in CK19-positive ductular reaction cells. Further investigation proved that the number of autophagosomes was higher in human fibrotic liver samples and the mouse CDE liver injury model than in normal liver tissue from humans and mice.

To investigate the correlation between autophagy and hepatic differentiation of CK19-positive HPCs, we established hepatic differentiation of HPCs in vitro, confirming that autophagy was highly activated in the progression of hepatic differentiation of HPCs. This result was similar to recent studies demonstrating that autophagy is involved in differentiation in different cells, including monocytes, satellite cells and neural stem cells (Zhang et al. 2012; Vázquez et al. 2012; García-Prat et al. 2016). In a subsequent study, we found that inhibition of autophagy by downregulation of Atg5 gene expression impaired hepatic differentiation of CK19-positive HPCs in vivo and in vitro, which is in line with previous investigations demonstrating that autophagy impairment inhibits the differentiation of stem cells, including glioma stem/progenitor cells, megakaryocytes and monocyte-macrophages (Zhao et al. 2010; Cao et al. 2015).

The Wnt/β-catenin signaling pathway is necessary for the differentiation of stem cells. Activation of Wnt/β-catenin signaling can promote the differentiation of stem cells (Shao et al. 2017; Huang et al. 2017). These results demonstrate that the Wnt/β-catenin pathway plays a vital role in regulating the differentiation of stem cells. However, the role of Wnt/β-catenin signaling in the hepatic differentiation of HPCs is unclear. We demonstrated that increased activation of Wnt/β-catenin signaling occurred during the hepatic differentiation of HPCs in vivo and vitro and that inhibiting autophagy by downregulating Atg5 gene expression suppressed activation of this pathway and then impeded the hepatic differentiation of HPCs; however, this phenomenon was reversed by overexpression of β-catenin. Although signaling cascades can reportedly modulate autophagy (Yang and Klionsky 2010), how autophagy selectively regulates the turnover of signaling molecules is largely unclear. Recent studies have revealed that autophagy stimulates the proliferation of porcine pancreatic stem cells and that this process is regulated by the canonical Wnt signaling pathway (Ren et al. 2016). Regardless, the correlation between autophagy and Wnt/β-catenin signaling during hepatic differentiation of HPCs remains unknown. In our study, we confirmed that inhibition of autophagy by downregulation of Atg5 gene expression suppressed the activation of Wnt/β-catenin signaling and impaired the hepatic differentiation of HPCs in vivo and vitro and that inhibition of β-catenin alone without inhibition of autophagy also impaired HPC differentiation. Overexpression of β-catenin rescued the impairment in the hepatic differentiation of HPCs induced by inhibition of autophagy. pGSK3β, a phosphorylated form of GSK3β, plays an important role in regulating the activation of β-catenin. In our study, Wnt/β-catenin signaling pathway was activated when pGSK3β was decreased, but that it was inhibited when pGSK3β was increased, similar to the findings of previous studies (Mao et al. 2018; Zheng et al. 2013). Upon further investigation, we found that P62 could form a complex with pGSK3β. Although the study of the mechanism underlying the hepatic differentiation of HPCs is not sufficiently comprehensive, the results are helpful to further understand the mechanism of hepatic differentiation of HPCs.

In summary, we found that autophagy promoted the hepatic differentiation of HPCs by regulating Wnt/β-catenin signaling in vivo and vitro. These findings will further increase our understanding of the molecular mechanism underlying HPC hepatic differentiation.
